# Socioeconomic Status and Foodborne Pathogens in Connecticut, USA, 2000–2011^1^

**DOI:** 10.3201/eid2109.150277

**Published:** 2015-09

**Authors:** Bridget M. Whitney, Christina Mainero, Elizabeth Humes, Sharon Hurd, Linda Niccolai, James L. Hadler

**Affiliations:** Yale School of Public Health, New Haven, Connecticut, USA

**Keywords:** Shiga toxin–producing *Escherichia coli*, STEC, *Escherichia coli* O157, bacteria, hemolytic uremic syndrome, Salmonellae, salmonellosis, socioeconomic status, SES, enteric infections, epidemiology, Connecticut, United States

## Abstract

Foodborne pathogens cause >9 million illnesses annually. Food safety efforts address the entire food chain, but an essential strategy for preventing foodborne disease is educating consumers and food preparers. To better understand the epidemiology of foodborne disease and to direct prevention efforts, we examined incidence of *Salmonella* infection, Shiga toxin–producing *Escherichia coli* infection, and hemolytic uremic syndrome by census tract–level socioeconomic status (SES) in the Connecticut Foodborne Diseases Active Surveillance Network site for 2000–2011. Addresses of case-patients were geocoded to census tracts and linked to census tract–level SES data. Higher census tract–level SES was associated with Shiga toxin–producing *Escherichia coli*, regardless of serotype; hemolytic uremic syndrome; salmonellosis in persons ≥5 years of age; and some *Salmonella* serotypes. A reverse association was found for salmonellosis in children <5 years of age and for 1 *Salmonella* serotype. These findings will inform education and prevention efforts as well as further research.

## Abstract 2

Diseases were associated with socioeconomic status and age of patients and with serotype of organism.

Foodborne diseases cause considerable illness, hospitalization, and death in the United States. Each year, an estimated 9.4 million illnesses, 56,000 hospitalizations, and 1,351 deaths can be attributed to the consumption of food products contaminated by 31 major pathogens (*1*). *Salmonella* and Shiga toxin–producing *Escherichia coli* (STEC) are leading bacterial causes of foodborne illness in the United States and result in a combined estimated 1.2 million cases of gastrointestinal illness, ≈22,000 hospitalizations, and 400 deaths per year (*1*). 

Food safety is a high priority in the United States (*2*). Although food safety efforts address the entire food chain from production to the retail level (*3*), these processes do not guarantee that food products, especially uncooked fresh foods, are free from potentially pathogenic bacteria. Therefore, an essential strategy for preventing foodborne disease involves educating food preparers and consumers about preventive measures that can be taken in food handling, cooking, and selection of foods to eat (*4*).

Despite regulatory efforts to improve food supply safety, the incidence of illnesses caused by some foodborne pathogens, including *Salmonella*, has changed little in recent years (*5*). Other than what is known about foodborne illness in younger and older age groups, little is known about which demographic groups in the United States are at highest risk for *Salmonella* or STEC infection and which groups should be targeted for educational efforts. Demographic data other than age and sex, such as income and education level, are not usually available through routine surveillance of illnesses from these infections.

An approach rarely used to identify demographic groups at high risk for bacterial foodborne infections is to examine incidence by area-based socioeconomic status (ABSES) measures. Surveillance data usually include street addresses of residences of persons diagnosed with foodborne infections, making use of ABSES possible. Census tract–level poverty, in particular, is a validated ABSES measure recommended by the Public Health Disparities Geocoding Project on the basis of a series of exhaustive studies (*6*). A previous Connecticut study assessing incident *Campylobacter* data that used census tract–level poverty found that adults and children >10 years of age who lived in census tracts where <5% of residents lived below the federal poverty level had twice the risk for campylobacteriosis of those living in census tracts where >20% lived below the federal poverty level (*7*). By contrast, children <10 years of age who lived in the lowest SES census tracts had a 1.4-fold higher risk for campylobacteriosis than those living in the highest SES census tracts (*7*). A study in Denmark, where individual SES data were available, found that *Campylobacter* and *Salmonella enterica* serotype Enteritidis were associated with high SES but found no association with *S. enterica* ser. Typhimurium or STEC (*8*). A study that used ABSES to examine *Salmonella* incidence in Michigan, USA, found that persons living in census block groups with high education levels had a higher incidence of *Salmonella* infection than persons living in block groups with lower education levels (*9*).

Our study sought to describe the incidence of *Salmonella* (in general and for leading serotypes) and of STEC (O157, non-O157, and hemolytic uremic syndrome [HUS]) for 2000–2011 by census tract–level SES and to assess whether findings changed over time. Our goal was to help direct public health educational efforts to decrease illness from these foodborne pathogens.

## Methods

### Case Identification and Data Collection

This analysis used data from the Foodborne Diseases Active Surveillance Network (FoodNet) in Connecticut for all incident cases of *Salmonella* and STEC infection with onset during 2000–2011. *Salmonella*, *Escherichia coli* O157:H7 gastroenteritis, Shiga toxin–related disease, and HUS are reportable by physicians and laboratories in Connecticut. All isolates of *Salmonella* and *E. coli* O157:H7 and broths from positive Shiga toxin test results are sent to the Connecticut Department of Public Health (DPH) Laboratory for confirmation, isolation (Shiga toxin–positive broths), and serotyping (*10*). Demographic information was abstracted from the case report form, including street address, age, and sex of case-patients.

### Geocoding of Case-Patients and Census Tract–Level SES

Street addresses were geocoded in ArcGIS 10.1 (Esri, Redlands, CA, USA) by using Topologically Integrated Geographic Encoding and Referencing shape files from the US Census Bureau and either a US street locator or a North American address locator. If automatic ArcGIS settings were unsuccessful, interactive geocoding was performed. Case-patients whose addresses were geocoded were matched to census tracts by using a spatial join in ArcGIS: those for 2000–2005 were spatially joined to census tracts by using the 2000 Census; those for 2006–2011 were joined to census tract designations from the 2010 Census. Census tract-specific SES data for percentage of the population living below the federal poverty line were taken from the 2000 Census for case-patients for 2000–2005 (*11*) and from the 2006–2010 American Community Survey for case-patients for 2006–2011 (*12*). Census tract–level SES was categorized into 4 groups based on percentage of residents living below the federal poverty line: <5%, 5%–9.9%, 10%–19.9%, and >20% (*6*).

### Statistical Analysis

Statistical analysis was limited to case-patients whose address could be geocoded and successfully linked to a census tract. During 2000 and 2010, data were missing for 7 (<1%) census tracts, so they could not be assigned to a poverty category, but no case-patients resided in these tracts. All case-patients were aggregated into 3 age categories on the basis of similar age-specific incidence rates: 0–4, 5–9, and >10 years of age for *Salmonella* cases and 0–4, 5–17, and ≥18 years of age for STEC cases. Within age groups, case-patients in each SES category were aggregated to determine the numerator for each group. Denominators by age group for each census tract were obtained from the 2000 Census for case-patients for 2000–2005 and from the 2010 Census for case-patients for 2006–2011 (*13*,*14*). Denominators were similarly aggregated to create age-specific denominators by SES category. Crude, age-adjusted, and age-specific incidence rates (IRs) per 100,000 person-years for all *Salmonella*, STEC, and HUS case-patients were calculated for each SES category. Age-adjusted rates were calculated by using direct standardization with weights taken from the US 2000 Standard Populations (*15*). Age-adjusted IRs per 100,000 person-years were also calculated for each of the 9 leading *Salmonella* serotypes and for O157 and non-O157 STEC subtypes. Incidence rate ratios (IRRs) were calculated for age-adjusted rates for SES categories by using the <5% poverty group as the reference. IRRs were calculated separately for all *Salmonella*, STEC, and HUS case-patients and for the leading *Salmonella* serotypes and O157 and non-O157.

Associations between *Salmonella*, STEC, and HUS incidence and census tract–level SES were examined by using χ^2^ tests for trend to test the statistical significance of the gradients among the 4 categories. All analyses were conducted by using SAS 9.3 (SAS Institute Inc., Cary, NC, USA) except χ^2^ tests for trend, which were calculated by using Epi Info 2000 (Centers for Disease Control and Prevention, Atlanta, GA, USA).

## Results

Overall, the SES category with the most persons in Connecticut was the highest SES group, <5% below the poverty line (55.2% of the population during 2000–2005 and 48.9% during 2006–2011). The lowest SES group, >20% below the federal poverty level, was the smallest for both periods (10.5% and 12.4% of the population, respectively).

### *Salmonella* Infection

During 2000–2011, a total of 5,484 case-patients with *Salmonella* infection confirmed by culture were reported to the Connecticut DPH and FoodNET. Of these, 5,204 (94.9%) were matched to a census tract. Case-patients that could not be matched did not differ from matched case-patients by age group or sex; however, a higher percentage of case-patients could not be matched during the earlier years of surveillance than in the later years (8.1% in 2000–2002 vs. 3.0% in 2009–2011; p<0.001). Serotype information was available for all but 1 case-patient. The most frequently observed serotypes were Enteriditis (n = 1,350, 25.9%), Typhimirium and its variants (n = 1,000, 19.2%), Newport (n = 353, 6.8%), and Heidelberg (n = 178, 3.4%). The overall *Salmonella* IR for 2000–2011 was 12.43 cases per 100,000 person-years. Younger children had the highest IRs; children 0–4 years of age had a 3.47-fold higher rate and children 5–9 years of age a 1.46-fold higher rate of *Salmonella* infection than those >10 years of age (Table 1). The *Salmonella* IR in the last 6 years of the study period (2006–2011) was 5.6% higher than for the first 6 years (12.75 vs. 12.08; p = 0.05). No statistical differences were found in *Salmonella* incidence by sex.

**Table 1:** Incidence of salmonellosis by age group and census tract–level SES, Connecticut, USA, 2000–2011*

Age group	Census tract–level SES, % living below poverty level					p value†
Total	<5	5–9.9	10–19.9	≥20
All ages						
Case-patients, no.	5,204	2,797	1,078	772	557	<0.001
Person-years	41,877,972	21,746,820	8,823,930	6,478,176	4,802,394	
Rate‡	12.43	12.86	12.22	11.92	11.60	
0–4 y						
Case-patients, no.	931	429	170	185	147	0.058
Person-years	2,552,700	1,238,688	508,140	432,696	373,176	
Rate‡	36.47	34.63	33.46	42.76	39.38	
5–9 y						
Case-patients, no.	431	262	66	57	46	0.029
Person-years	2,800,290	1,484,166	539,844	420,924	355,356	
Rate‡	15.39	17.65	12.23	13.54	12.94	
≥10 y						
Case-patients, no.	3,842	2,106	842	530	364	<0.001
Person-years	36,524,982	19,023,966	7,775,946	5,624,556	4,073,862	
Rate‡	10.52	11.07	10.83	9.42	8.94	

*A total of 2,221 persons were living in census tracts unable to be classified by SES level. SES, socioeconomic status. †By χ^2^ test for trend. ‡No. cases/100,000 person-years.

Higher census tract–level SES was associated with higher crude *Salmonella* incidence for all 12 years of data combined (Table 1), for each of the two 6-year periods (data not shown), and among children 5–9 and >10 years of age (Table 1). A reverse association was found for the 0–4 year age group. The highest *Salmonella* IRs occurred in the 2 lowest SES groups (>20% = 39.38 and 10%–19.9% = 42.76); the lowest IRs occurred in the 2 highest SES groups (5%–9.9% = 33.46 and <5% = 34.63) (Table 1).

The overall association of higher *Salmonella* incidence with higher SES was amplified after age-adjustment; the lowest SES group had a 0.86-fold lower IRR than the highest SES group (13.18 vs. 11.34; p = 0.01; Table 2). Notable differences in age-adjusted rates also occurred for individual *Salmonella* serotypes. Although the same association of higher incidence and higher census tract–level SES was found for serotypes Enteriditis (IRR 0.67 lowest vs. highest SES groups, p <0.001), Newport (IRR 0.53, p = 0.002), and Montevideo (IRR 0.52, p = 0.046), a reverse gradient of higher incidence and lower census tract–level SES was found for serotype Heidelberg (IRR 1.94, p = 0.001; Table 2). When *S. enterica* ser. Heidelberg was examined by age group, only children 0–4 years of age had a significant association with census tract–level SES; this group of children in the lowest SES group had a 4.98-fold higher IRR than this age group in the highest SES group (p<0.001). No other serotype had an association with census tract–level SES, including Typhimurium, the second most common serotype.

**Table 2:** Age-adjusted incidence rates and age-adjusted rate ratios of salmonellosis and 9 leading *Salmonella enterica* serotypes by census tract–level SES, Connecticut, USA, 2000–2011*

*Salmonella* serotype	Census tract–level SES, % living below poverty level				p value†
	<5	5–9.9	10–19.9	≥20	
Total,, N = 5,204					0.012
Age-adjusted IR	13.18	12.50	12.02	11.34	
Age-adjusted IRR	1.00	0.95	0.91	0.86	
Enteriditis,, n = 1,350					<0.001
Age-adjusted IR	3.72	3.09	2.40	2.51	
Age-adjusted IRR	1.00	0.83	0.65	0.67	
Heidelberg, n = 178					0.001
Age-adjusted IR	0.35	0.46	0.47	0.68	
Age-adjusted IRR	1.00	1.31	1.34	1.94	
Montevideo, n = 98					0.046
Age-adjusted IR	0.27	0.28	0.16	0.14	
Age-adjusted IRR	1.00	1.04	0.59	0.52	
Newport, n = 353					0.002
Age-adjusted IR	0.94	0.86	0.77	0.50	
Age-adjusted IRR	1.00	0.91	0.82	0.53	
Oranienburg, n = 109					0.472
Age-adjusted IR	0.29	0.23	0.27	0.23	
Age-adjusted IRR	1.00	0.79	0.93	0.79	
Saintpaul, n = 130					0.053
Age-adjusted IR	0.29	0.29	0.29	0.47	
Age-adjusted IRR	1.00	1.00	1.00	1.62	
I 4,[5],12:i:-, n = 134					0.585
Age-adjusted IR	0.33	0.27	0.40	0.36	
Age-adjusted IRR	1.00	0.82	1.21	1.09	
Thompson, n = 96					0.441
Age-adjusted IR	0.26	0.20	0.22	0.21	
Age-adjusted IRR	1.00	0.77	0.85	0.81	
Typhimurium, n = 1,000					0.913
Age-adjusted IR	2.41	2.53	2.57	2.40	
Age-adjusted IRR	1.00	1.05	1.07	1.00	

*IR, incidence rate; IRR, incidence rate ratio, SES, socioeconomic status. Age-adjusted IRs calculated/100,000 persons; Reference category for age-adjusted IRRs is <5% poverty. †By χ^2^ test for trend.

### STEC

During 2000–2011, a total of 764 case-patients with STEC confirmed by culture were reported to the Connecticut DPH and FoodNET. Of these, 744 (97.4%) were matched to a census tract. Those that could not be matched did not differ from matched cases. The most frequently observed serotypes were STEC O157 (n = 471, 63.3%), O103 (n = 55, 7.4%), O111 (n = 51, 6.0%), O26 (n = 42, 5.6%), and O45 (n = 26, 3.5%). The overall STEC IR for 2000–2011 was 1.78 case-patients/100,000 person-years. Younger children had higher IRs than persons >18 years; children 0–4 years of age had a 4.81-fold higher rate of STEC than those >18 years of age, and those 5–17 years of age had a 3.96-fold higher rate of STEC than those >18 years of age. The STEC IR in the last 6 years of the study period (2006–2011) was 16% lower than in the first 6 years (1.65 vs. 1.91; p = 0.05). Female patients across all age groups had a 1.31-fold higher IR for STEC than male patients (p<0.001).

Higher census tract–level SES was associated with higher crude STEC incidence for all 12 years of data combined (Table 3), within each 6-year period (data not shown), and for each of the 3 age groups (Table 3). The association was stronger for those >5 years of age (IRR for highest SES vs. lowest SES group = 6.21 for persons 5–17 years of age and 4.06 for persons >18 years of age; p<0.001 for each) and lower for children >4 years of age (IRR = 1.73; p = 0.054; Table 3).

**Table 3:** Incidence of STEC by age group and census tract–level SES, Connecticut, USA, 2000–2011*

All STEC, N = 744	Census tract–level SES, % living below poverty level					p value†
Total	<5	5–9.9	10–19.9	≥20
All ages						<0.001
Case-patients, no.	744	498	138	77	31	
PY	41,877,972	21,746,820	8,823,930	6,478,176	4,802,394	
Rate^‡^	1.78	2.29	1.56	1.19	0.65	
0–4 y						
Case-patients, no.	124	69	25	18	12	0.054
PY	2,552,700	1,238,688	508,140	432,696	373,176	
Rate^‡^	4.86	5.57	4.92	4.16	3.22	
5–17 y						
Case-patients, no.	296	220	49	19	8	<0.001
PY	7,399,518	3,977,634	1,435,740	1,088,280	897,636	
Rate‡	4.00	5.53	3.41	1.75	0.89	
≥18 y						
Case-patients, no.	324	209	64	40	11	<0.001
PY	31,925,754	16,530,498	6,880,050	4,957,200	3,531,582	
Rate^‡^	1.01	1.26	0.93	0.81	0.31	

*PY, person-years; SES, socioeconomic status; STEC, Shiga toxin–producing *Escherichia coli*. Includes 2,221 persons living in census tracts unable to be classified by socioeconomic level. †p-value is for χ^2^ test for trend. ‡Rate = number of cases/100,000 person-years.

As with *Salmonella*, the overall association of higher STEC incidence with higher census tract–level SES was stronger after age adjustment (Table 4); the IRR for the lowest versus the highest SES group was 0.26 (p<0.001; Table 4). This association occurred for E*. coli* O157 and for non-O157 STEC (IRRs of 0.24 and 0.29, respectively; p<0.001 for each; Table 4). We also examined the age-adjusted incidence and its relationship with census tract–level SES for the much smaller number of HUS cases (n = 49). The same association of higher HUS incidence with higher SES was found. The IRR for the lowest versus the highest SES census tracts was 0.25 (p = 0.007; Table 4).

**Table 4:** Age-adjusted incidence rates and age-adjusted rate ratios of STEC O157, non-O157, and HUS by census tract–level SES, Connecticut, USA, 2000–2011*

STEC category	Census tract–level SES, % living below poverty level				p value†
	<5	5–9.9	10–19.9	≥20	
All STEC, N = 744					<0.001
Age-adjusted IR	2.36	1.67	1.22	0.62	
Age-adjusted IRR	1.00	0.71	0.52	0.26	
STEC O157, n = 471					<0.001
Age-adjusted IR	1.48	1.20	0.70	0.36	
Age-adjusted IRR	1.00	0.81	0.47	0.24	
STEC non-O157, n = 273					<0.001
Age-adjusted IR	0.89	0.48	0.52	0.26	
Age-adjusted IRR	1.00	0.54	0.58	0.29	
HUS, n = 49					<0.001
Age-adjusted IR	0.16	0.17	0.03	0.04	
Age-adjusted IRR	1.00	1.04	0.19	0.25	

*IR, incidence rate; IRR, incidence rate ration; HUS, hemolytic uremic syndrome; SES, socioeconomic status; STEC, Shiga toxin–producing *Escherichia coli*. Age-adjusted IRs calculated/100,000 persons; Reference *category* for age-adjusted IRRs is <5% poverty. †By χ^2^ test for trend.

## Discussion

Few studies are available that examine the relationship between foodborne disease incidence and socioeconomic status. Our study showed the following key findings: 1) STEC disease, whether caused by O157 or non-O157 serotypes or whether manifested as HUS, was uniformly associated with high census tract SES; 2) salmonellosis in persons 5–9 and >10 years of age was associated with high census tract SES, whereas salmonellosis in children <5 years of age was associated with low census tract SES; and 3) different *Salmonella* serotypes had different associations with census tract SES, with serotypes Enteriditis, Newport, and Montevideo associated with high census-tract SES, serotype Heidelberg associated with low census-tract SES, and serotype Typhimurium having no association. These findings provide additional information about the epidemiology of these foodborne diseases and should inform efforts to reduce their incidence.

Our findings study are similar to those found in a study of campylobacteriosis in Connecticut during the same period (*7*): higher disease incidence among those living in higher SES census tracts. A previous study in Michigan found that *Salmonella* incidence increased with higher education and income levels (*9*). An analysis of recent FoodNet data by race/ethnicity showed that overall STEC rates were highest for whites (a surrogate for higher SES) and lowest for blacks (a surrogate for lower SES) (*16*). The current study results were consistent with results from these studies that examined surrogates for SES.

Several explanations have been suggested to explain why persons in higher SES census tracts might have higher incidence of *Salmonella* and STEC infections and HUS (and *Campylobacter* infection), compared with persons in lower SES census tracts. A commonly proposed reason is that those living in areas with higher census tract–level SES might have increased access to care and might be more likely to submit specimens, regardless of disease severity, whereas those in lower socioeconomic groups might seek care or diagnostic testing only when illness is serious or prolonged (*8*,*9*,*17*). Several lines of evidence argue against this explanation in the United States. For 2000–2003, FoodNET assessed factors associated with seeking medical care and submitting a fecal specimen among persons with acute diarrheal illness and found that ≈20% of persons with acute diarrheal diseases sought medical care, 19% of whom submitted a fecal specimen (*18*). The analysis found that a household income <$25,000 was associated with seeking medical care (*18*). This association of lower income with seeking medical care for diarrheal illness, the uniform trend of increasing incidence from lowest to highest SES group, and the opposite association for some *Salmonella* serotypes indicate that medical-seeking behavior is not a major explanatory factor for our results. Furthermore, HUS, a disease almost always requiring hospitalization and thus less subject to potential health-seeking bias, had the same association with higher SES as did milder forms of STEC infection.

The more likely explanation for these findings is that SES affects the prevalence of known high-risk factors, such as international travel, consumption of high-risk food items (e.g., raw fruits and vegetables and undercooked meat), and eating at restaurants (*16*). That is, high SES itself is not a risk factor but rather a surrogate for certain high-risk behaviors. An analysis of the Connecticut portion of 3 FoodNet population surveys during 2000–2007 found that higher-income residents were more likely than lower-income residents to have traveled internationally, eaten in restaurants, and eaten chicken in the previous 7 days (*17*). An analysis of population survey data from the 2006–2007 Connecticut FoodNet found that residents with higher SES ZIP codes were more likely than those with lower SES ZIP codes to have eaten fresh hamburger at home that was pink, to have consumed salad containing lettuce or greens, and to have traveled internationally in the previous 7 days (J. Wagner, unpub. data). Studies outside the Connecticut FoodNET have had similar results. Several studies have found that contaminated raw fruits and vegetables are a growing source of outbreaks in the United States and have increased in both numbers and proportions of all reported foodborne outbreaks (*19*,*20*). A recent food attribution study attributed 32% of all bacterial foodborne illnesses, of which *Salmonella* and STEC make up a large proportion, to produce commodities, including fruits, nuts, and vegetables of the fungi, leafy, root, sprout, and vine-stalk variety (*21*). High SES is associated with more fruit and vegetable consumption, which may be an explanatory factor for our findings. A study in the United States found that higher neighborhood SES was positively associated with fruit and vegetable intake and that an increase of 1 SD in neighborhood SES was associated with 2 additional servings of fruit and vegetables per week (*22*). Low SES communities often have access to fast food and prepackaged food but lack adequate supermarkets, which causes limited access to fresh fruits and vegetables (*23*,*24*). In addition, ≈40% of adolescents from low SES backgrounds have less than daily consumption of fruits and vegetables (*25*). A study addressing fruit and vegetables as vehicles for transmission of foodborne pathogens found that the association of *Salmonella* with fresh produce appears to be serotype-specific because of adhesion mechanisms in some serotypes (*26*), a finding that may partly explain why the association of higher SES with higher incidence was seen only among some serotypes of *Salmonella*.

In contrast to the findings in adults, children <5 years of age who live in low SES census tracts were more likely than those living in high SES census tracts to have *Salmonella* infection. In addition, all persons with *S. enterica* ser. Heidelberg infection were more likely to live in a low SES census tract. These findings are novel: the Michigan study did not look at age, and no reported studies in the United States have systematically examined the relationship between census tract–level SES and *Salmonella* serotype incidence. However, the previously published Connecticut campylobacteriosis study had similar findings: children living in low SES census tracts had the highest incidence (*7*,*27*). Several studies have shown that young children in low SES circumstances are more likely to be exposed to raw meat–contaminated surfaces inside and outside the home (e.g., in shopping carts in grocery stores) (*16*,*17*). Also, different *Salmonella* serotypes are associated with different food items (*21*). *S. enterica* ser. Heidelberg has been associated with the consumption of eggs and poultry (*28*–*30*), and a study assessing *Salmonella* prevalence in 6 commodities at point of processing found high prevalence of *S. enterica* ser. Heidelberg in chicken and turkey (*31*). Possibly, more eggs and poultry are consumed in lower SES groups than in higher SES groups.

Our findings have several implications for risk communication and research. Efforts could be made to increase awareness among persons in high SES groups about their relatively high risk for STEC and *Salmonella* infection and about what actions they can take to reduce risk. Education campaigns about high-risk foods other than meat and about the importance of properly handling produce could be run in publications with a higher SES target audience. Regarding research, several considerations need to be explored further. Whether our findings in Connecticut are representative of STEC and salmonellosis incidence nationwide is unknown. The specific reasons behind SES-related risk for foodborne illness still need to be made clear, including for each of the leading *Salmonella* serotypes. For young children with salmonellosis, potential intervention points to reduce exposure inside and outside the home need to be identified. It remains unclear why children living in areas of higher SES are more at risk for STEC infection and HUS than children living in lower SES areas. These unanswered questions need to be investigated so that effective consumer-level interventions can be developed.

This study has several limitations. First, it was limited to data from Connecticut, and findings might not be generalizable to other states. Second, SES information was unavailable for 7 census tracts; however, they accounted for <1% of all census tracts in Connecticut. Third, although the data came from active surveillance and are therefore complete within the context of laboratory-confirmed disease, not all persons with gastrointestinal illness seek medical care or have diagnostic testing (*1**,**18*), so these data are underestimates of the true incidence of STEC and salmonellosis. Fourth, this study used SES measured at the census tract, not individual level; the results of this analysis should be understood and interpreted within this context. Finally, we did not look at the consistency of the associations of incidence with SES for different adult age groups but assumed they were similar.

Public health infrastructure objective 7.3 of Healthy People 2020 stresses the need to “increase the proportion of population-based Healthy People 2020 objectives for which national data are available by socioeconomic status” (*32*). This study provides additional evidence that area-based SES measures such as census tract–level poverty, especially if other SES measures are unavailable, can be useful for describing the incidence of foodborne illnesses. Our findings show differences in STEC and *Salmonella* serotype-specific incidence by census tract–level SES. The findings suggest direction for risk communication efforts and for additional studies to explain the differences and to facilitate additional education and outreach activities.
